# Klotho preservation via histone deacetylase inhibition attenuates chronic kidney disease-associated bone injury in mice

**DOI:** 10.1038/srep46195

**Published:** 2017-04-07

**Authors:** Wenjun Lin, Yanning Li, Fang chen, Shasha Yin, Zhihong Liu, Wangsen Cao

**Affiliations:** 1 National Clinical Research Center of Kidney Diseases, Jinling Hospital, Nanjing University School of Medicine, Nanjing, China; 2 Nanjing University School of Medicine, Jiangsu Key lab of molecular Medicine, Nanjing, China

## Abstract

Bone loss and increased fracture are the devastating outcomes of chronic kidney disease-mineral and bone disorder (CKD-MBD) resulting from Klotho deficit-related mineral disturbance and hyperparathyroidism. Because Klotho down-regulation after renal injury is presumably affected by aberrant histone deacetylase (HDAC) activities, here we assess whether HDAC inhibition prevents Klotho loss and attenuates the CKD-associated bone complication in a mouse model of CKD-MBD. Mice fed adenine-containing diet developed the expected renal damage, a substantial Klotho loss and the deregulated key factors causally affecting bone remodeling, which were accompanied by a marked reduction of bone mineral density. Intriguingly, administration of a potent HDAC inhibitor trichostatin A (TSA) impressively alleviated the Klotho deficit and the observed alterations of serum, kidney and bone. TSA prevented Klotho loss by increasing the promoter-associated histone acetylation, therefore increasing Klotho transcription. More importantly the mice lacking Klotho by siRNA interference largely abolished the TSA protections against the serum and renal abnormalities, and the deranged bone micro-architectures. Thus, our study identified Klotho loss as a key event linking HDAC deregulation to the renal and bone injuries in CKD-MBD mice and demonstrated the therapeutic potentials of endogenous Klotho restoration by HDAC inhibition in treating CKD and the associated extrarenal complications.

Renal osteodystrophy and subsequent bone fracture are common complications of chronic kidney diseases (CKD), now classified as CKD-mineral and bone disorder (CKD-MBD)^1^, which is characterized by severe renal injury, disturbed mineral and hormone metabolisms accompanied by cardiovascular and bone complications. The low glomerular filtration rate associated with early CKD development retains excessive phosphorus that causes progressive rise of osteocyte-derived fibroblast growth factor-23 (FGF23) and parathyroid hormone (PTH) production, which adversely affect bone remodeling and resorption eventually ensuring osteoporotic bone complications^2^. The medications aiming at minimizing phosphorus retention through dietary therapy or alternative vitamin D supplementation improved the abnormal metabolism of minerals and hormones^3^. On the other hand, novel strategies modulating endogenous renal protective proteins might provide additional protections against the pathological processes. Recently a newly identified renal protein Klotho is found to be a key regulator of mineral metabolisms and kidney homeostasis^4^,^5^ and a potential target for treating the renal and bone injuries of CKD-MBD.

Klotho was originally identified as an antiaging protein highly expressed in renal tubule epithelial cells^4^,^6^. Mice lacking Klotho display accelerated aging phenotypes and the renal and extrarenal manifestations resembling patients with CKD-MBD^7^. In addition, the mice showed the low bone formation and resorption activities^8^,^9^, suggesting that Klotho can directly affect bone remodeling. Klotho is a membrane protein mainly functioning as a cofactor for FGF23 (fibroblast growth factor −23)^10^, through which it regulates phosphate and vitamin D metabolisms controlling the mineral levels in the body. A soluble form of Klotho, generated by differential splicing or ectodomain cleavage from the membrane-bound Klotho and released into blood, cerebral fluid and urine, possesses β-glucuronidase activity and regulates various cell receptors and ion transporters critically protecting kidney and bone functions^11^,^12^,^13^. Klotho is sensitive to various acute or chronic renal injuries and declined early in renal patients and experimental animals of CKD^14^,^15^, whereas transgenic Klotho over-expression, exogenous Klotho supplementation or the maintaining Klotho via re-activating endogenous Klotho protects against the renal damage and functional loss in CKD animals^16^,^17^,^18^, suggesting that Klotho is a remarkable renal protector with therapeutic potentials. Klotho suppression after renal injury reportedly involves transcription factor-mediated transcriptional repression and epigenetic DNA or protein modifications^19^,^20^ among them deregulated histone deacetylase (HDAC) activities seem to be an important but less explored mechanism that promotes the pathological Klotho down-regulation.

Protein acetylation modifications of histone or non-histone proteins, catalyzed by two groups of enzymes of opposite functions - histone acetyl-transferase and HDAC, are emerging as fundamental mechanisms controlling renal physiology and pathogenesis^21^,^22^,^23^,^24^. Aberrant HDAC activities are associated with various acute or chronic kidney diseases such as diabetic nephropathy, CKD and polycystic kidney disease in animal studies, in which HADC inhibition significantly delayed the pathological processes^25^,^26^,^27^,^28^,^29^, but the underlying molecular mechanisms and the key effecter genes involved are incompletely understood. Recent studies reported that Klotho suppression after acute kidney injury involved the HDAC1/2-associated pro-inflammatory signaling^20^ and Klotho repressions incurred by the promoter hypermethylation in patients and animal studies of CKD^30^,^31^ are presumably mediated by a transcription repression mechanism requiring HDAC activities, raising the possibility that inhibition of HDAC might relieve Klotho repression and restore its renal protective capacities.

In this study we seek to investigate whether HDAC inhibition prevents Klotho suppression and its functional significances in a mouse model of adenine-induced CKD-MBD. Our study will clarify whether Klotho is a key gene involved in the HDAC aberration-associated renal and bone injuries and bring new insights into the Klotho-targeted therapies in treating CKD and the related extrarenal complications.

## Results

### HDAC inhibition prevents CKD-associated renal and bone damage in adenine-fed mice

**Figure 1:**
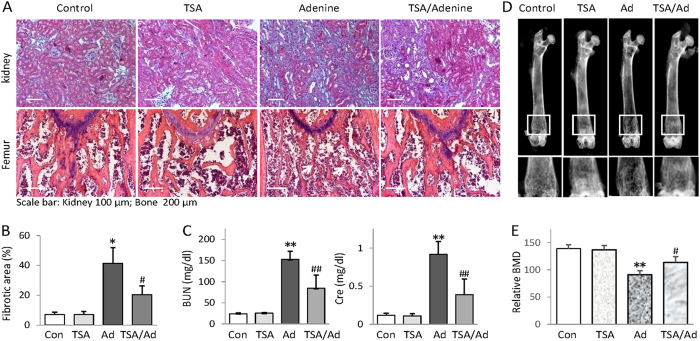
HDAC inhibition prevents CKD-associated renal and bone damage in adenine-fed mice. (**A**) Representative Masson’s trichrome staining of kidney sections and H&E stained femur sections from control, TSA, adenine and TSA-treated adenine mice (n = 6 in each group, 6 weeks). (**B**) Semi-quantifications of renal interstitial fibrosis (the percentage of blue-colored cortex area over the whole cortex field from Masson’s trichrome-stained sections) from all mice. (**C**) Average levels of serum blood urea nitrogen (BUN) and Creatinine (Cre). The quantifications were based on all mice tested. (**D**) Representative femur radiographs from Control, TSA, adenine and TSA-treated adenine mice. The lower panels are the enlarged views of framed images above (**E**) Quantifications of bone density of Fig. 1D from all mice. Data are presented as the mean ± SD. *P < 0.05, **P < 0.01 versus control; ^#^*P* < 0.05, ^##^*P* < 0.01 versus adenine mice.

To gain insights into the therapeutic efficacy of HDAC inhibition on CKD-associated bone complication, we established an adenine-fed mouse model of CKD-MBD^32^,^33^,^34^. We divided mice into control, TSA, adenine, and TSA-treated adenine groups and found that the kidneys from adenine mice fed 6 weeks developed extensive renal pathological changes such as renal tubular atrophy and interstitial fibrosis (Fig. 1A upper panel and Fig. 1B). In addition, adenine mice had marked increases of blood urine nitrogen (BUN) and creatinine (Fig. 1C) - two major parameters indicating the low glomerular infiltration rate due to renal functional loss. The histological examination of adenine mice revealed the marked osteoporosis-like changes on distal femurs - the thinner and deranged trabeculae with increased lacuna (Fig. 1A, the white arrow heads in lower panel). The X-ray scanning showed the marked loss of bone mineral density (BMD) at the distal femurs (Fig. 1D,E). Impressively, TSA (trichostatin A), a potent general HDAC inhibitor frequently used in HDAC inhibition study, effectively reduced the renal tubule damage and fibrosis lesions, attenuated the increased BUN and creatinine, and significantly improved the bone pathological changes and BMD loss (Fig. 1A–E). These results clearly demonstrate that HDAC inhibition protects against the renal and bone injuries in adenine mice.

### HDAC inhibition attenuates the CKD-associated abnormalities of mineral, hormone and osteogenic markers

**Figure 2:**
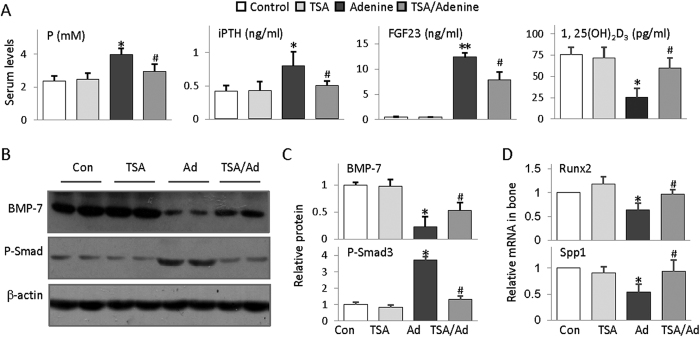
HDAC inhibition attenuates CKD-associated abnormalities of mineral, hormone and bone remodeling-related gene expression. (**A**) Average concentrations of serum phosphorus (P), intact parathyroid hormone (iPTH), FGF23 and 1,25-dihydroxyvitamin D3 (1,25 (OH)_2_D_3_ from Control, TSA, adenine and TSA-treated adenine mice of 6 weeks (n = 6). (**B**) Renal BMP-7 and phosphorylated Smad3 (P-Smad3) were examined from all mice by Western blotting (two randomly selected samples from each group were shown). Beta-actin (β-actin) served as loading control. (**C**) Quantifications of Figure 2B (n = 6 in each group). (**D**) Average levels of bone Runx2 and Spp1 mRNA examined by qRT-PCT (n = 6). The data are presented as mean ± SD. *P < 0.05, **P < 0.01 versus control; ^#^*P* < 0.05 versus adenine mice.

To gain further insight into the molecular basis of TSA protection of CKD-associated bone injury, we examined a number of key factors and osteogenic markers closely related to bone remodeling. Adenine mice displayed elevated serum phosphate, intact parathyroid hormone (iPTH) and FGF23, and decreased 1,25-dihydroxyvitamin D3 (1,25 (OH)_2_D_3_) (Fig. 2A) - the characteristic serological changes of CKD-MBD that causally related to bone damage. Bone morphogenesis protein-7 (BMP-7) is a kidney-enriched protein released into circulation and beneficially regulates renal function and bone remodeling, which is significantly reduced; while the phosphorylated Smad3, the active form of Smad3 mediating transforming growth factor-beta (TGFβ) signaling leading to myofibroblast trans-differentiation and extra cellular matrix protein expression, is dramatically induced in adenine mouse kidney (Fig. 2B,C). In addition, the femurs of adenine mice expressed reduced Runx2 (runt-related transcription factor 2) - a transcription factor critical for osteoblast differentiation and Spp1 (Secreted phosphoprotein 1) - an extracellular matrix protein essential for bone mass maintenance (Fig. 2D). Intriguingly TSA treatment significantly mitigated all these abnormalities, indicating that HDAC inhibition improves renal function and beneficially affects the CKD-associated bone remodeling.

### HDAC inhibition up-regulates renal Klotho

**Figure 3:**
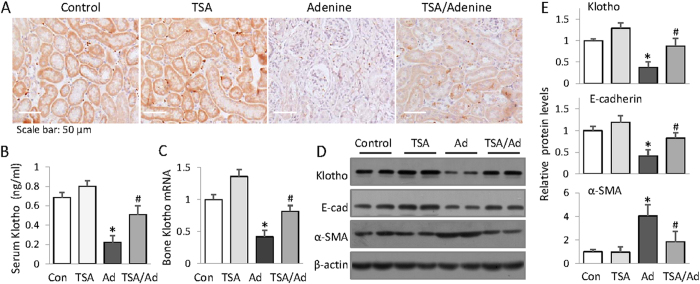
HDAC inhibition restores declined renal Klotho in adenine mice. (**A**) Immunohistochemical staining. The kidney sections from control, TSA, adenine and TSA-treated adenine mice (6 weeks, n = 6) were examined by immunohistochemical staining for renal Klotho expression. The representative figures from each group were shown. (**B**) Serum Klotho levels. The average concentrations of serum Klotho from control, TSA, adenine and TSA-treated adenine mice were measured by ELISA (n = 6). (**C**) Bone Klotho mRNA. The relative levels of Klotho mRNA were examined from control, TSA, adenine and TSA-treated adenine mouse femurs by qRT-PCR (n = 6). (**D**) Renal expressions of Klotho, α-SMA and E-cadherin were assayed by Western blotting from the mouse kidneys (2 randomly selected samples were shown). (**E**) Quantifications of Fig. 3D (n = 6). Data are presented as mean ± SD. *P < 0.05 versus control, ^#^*P* < 0.05 versus adenine mice.

Klotho is a key gene profoundly affecting mineral metabolism and bone remodeling, but reportedly suppressed after renal injury. We then decided to test whether HDAC inhibition affects Klotho expression. The results showed that Klotho levels in kidney, blood circulation and femur were all markedly reduced in adenine mice, as demonstrated by immunohistochemical and Western blotting examinations of kidney tissues (Fig. 3A,D and E), ELISA assay of mouse sera (Fig. 3B) and quantitative real time PCR measurement of mRNA in femur. (Fig. 3C). However TSA treatment impressively attenuated the Klotho reductions in all assays. In addition, the typical CKD-associated pathological alterations, such as the reduction of E-cadherin- an epithelial cell marker and the induction of α-SMA, the myofibroblast differentiation marker, were also attenuated by TSA treatment, indicating that HDAC inhibition effectively prevents Klotho loss, which might account for the renal and bone protections.

### HDAC inhibition up-regulates Klotho via reversing the promoter hypoacetylation and increasing Klotho transcription

**Figure 4:**
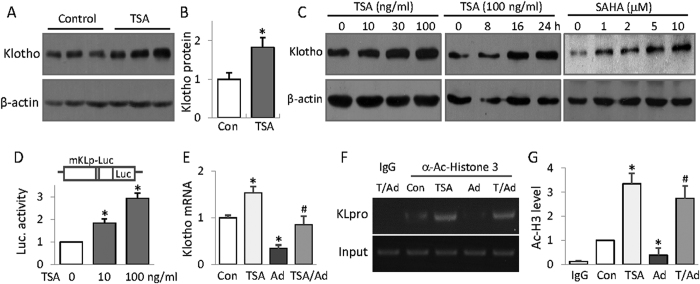
HDAC inhibition up-regulates Klotho via reversing the promoter hypoacetylation and increasing Klotho transcription. (**A**) TSA up-regulates Klotho in kidney. Kidney lysates from control and TSA-treated mice (6 weeks) were analyzed for Klotho protein levels by Western blotting (3 randomly selected samples from each group were shown). (**B**) Quantification of Fig. 3A. (**C**) HDAC inhibition dose and time-dependent up-regulation of Klotho in renal cells. HK2 cells were treated TSA of various doses (10, 30, or 100 ng/ml) or different times (100 ng/ml for 8, 16, and 24 h), or treated with SAHA of various amounts (1, 2, 5 and 10 μM) for 24 h, and then cell lysates were analyzed for Klotho protein expression by Western blotting. (**D**) Luciferase assay. HK2 cells were transfected with a mouse Klotho promoter reporter (mKLp-Luc) plus a renilla luciferase plasmid control for 20 h, and then TSA of two doses (10 and 100 ng/ml) was added to the cells for additional 24 h. The cell lysates were analyzed for luciferase activities, presented as the fold changes of the reporter luciferase activities divided by that of renilla control. (**E**) Klotho mRNA from mouse kidney. The average levels of Klotho mRNA from control (Con), TSA, adenine (Ad) and TSA-treated adenine mice (6 weeks, n = 6) were determined by qRT-PCR. (**F**) ChIP assay. Mouse kidney lysates from control, TSA, adenine and TSA-treated adenine mice were cross-linked and immune-precipitated with an anti-acetylated Histone3 antibody. The immune-precipitated DNAs were further PCR-amplified with primer sets specific for mouse Klotho promoter (KLpro). The genomic DNAs served as input control. The PCR products were analyzed on a 1.5% agarose gel and visualized under UC light. Representative results were shown. (**G**) Semi-quantification of Fig. 4F from all mice (n = 6 in each group). Data are presented as mean ± SD. Cell-bases assays were repeated three times and the representative results were shown. *P < 0.05 versus control; ^#^*P* < 0.05 versus adenine.

We next decided to further determine the epigenetic mechanism of Klotho up-regulation by HDAC inhibition. We found that TSA treatment increased the basal Klotho protein abundance in mouse kidney (Fig. 4A and B) and time- and dose-dependently increased Klotho protein levels in renal tubule HK2 cells (Fig. 4C). In order to ensure that Klotho up-regulation by TSA is indeed caused by HDAC inhibition, we also tested another general HDAC inhibitor SAHA (suberoylanilide hydroxamic acid) and found that SAHA also dose-dependently increase Klotho expression (Fig. 4C, right panel). To clarify whether TSA up-regulation of Klotho occurs at the gene transcription level, we transfected cells with a luciferase reporter plasmid containing 2 kb of mouse Klotho promoter and found that TSA dose-dependently increased the luciferase activities, thus, increasing the Klotho promoter transcription (Fig. 4D). Consistently, adenine mouse kidney exhibited reduced Klotho mRNA, but TSA treatment inhibited the reduction (Fig. 4E). Further we examined the histone acetylation status around Klotho promoter by ChIP (chromatin immunoprecipitation) assay. We precipitated the protein-cross-linked genomic DNA with a specific antibody to acetylated histone3, and then measured the amount of DNA associated with acetylated histone 3 by PCR using a primer set specific for Klotho promoter. The results showed that adenine mouse kidney displayed hypoacetylated histone3 on Klotho promoter, which is significantly alleviated by TSA treatment (Fig. 4F and G). Taken together these results suggest that HDAC inhibition recovered the Klotho suppression in adenine mouse kidney by increasing Klotho promoter acetylation and its transcription.

### Klotho is critical for the renal protection by HDAC inhibition

**Figure 5:**
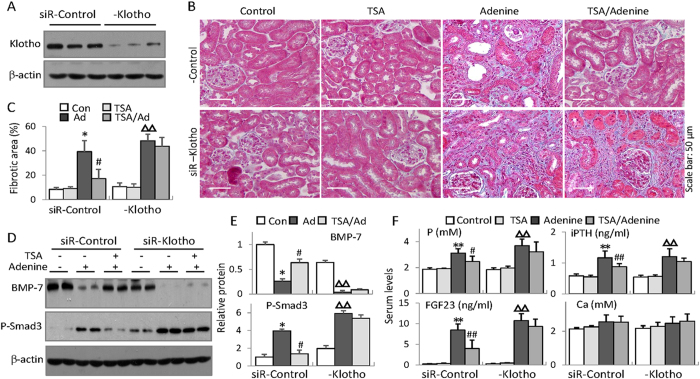
Klotho is critical for the renal protection by HDAC inhibition. Mice receiving siRNA-control or siRNA-Klotho underwent TSA, adenine or TSA plus adenine treatments for 6 weeks (n = 6 in each group). (**A**) Renal Klotho protein levels from siRNA-control or siRNA-Klotho-injected mice were examined (6 weeks, three randomly selected samples from each group were shown) by Western blotting. (**B**) Representative Masson’s trichrome staining of kidney sections from siRNA-control or siRNA-Klotho-injected control, TSA, adenine and TSA-treated adenine mice (6 weeks, n = 6 in each group). (**C**) Quantifications of renal interstitial fibrosis (the percentage of blue-colored cortex area over the whole cortex field from Masson’s trichrome-stained sections) from all mice in Fig. 5B. (**D**) Renal expressions of BMP-7 and phosphorylated Smad3 were examined by Western blotting (Two randomly selected samples were shown). (**E**) Quantifications of BMP-7 and P-Smad3 in Fig. 5D (n = 6). (**F**) Average serum levels of phosphorus (P), iPTH, FGF23 and Calcium (Ca), from all mice (n = 6). Data are presented as the mean ± SD. *P < 0.05, **P < 0.01 versus control, ^#^*P* < 0.05, ^##^*P* < 0.01 versus adenine treatment in siR-control mice; ^Δ^*P* < 0.05, ^ΔΔ^*P* < 0.01 versus control in siR-Klotho mice.

To determine the functional significance of Klotho restoration in the renal and bone protections by HDAC inhibition, we tested the effects of Klotho knockdown on the improved key factors and osteogenic markers related to bone injury by HDAC inhibition. We divided mice into two groups injected with either siRNA control or siRNA Klotho that effectively reduced renal Klotho (Fig. 5A) and then subjected mice to TSA and/or adenine treatment as before. The results showed that siRNA Klotho-treated mice displayed slight increase of renal fibrosis (Fig. 5B and C), phosphorylated Smad3 and reduced BMP-7 (Fig. 5D and E). Further, adenine feeding enhanced the alterations. Intriguingly, while TSA treatment reduced the renal damage and fibrosis and improved phosphorylated Smad3 and BMP-7 in siRNA control-injected mice, the protective effects were significantly reduced in siRNA Klotho-injected mice (Fig. 5A–E). Similarly TSA improvements of the increased serum phosphorus, iPTH and FGF23 were also significantly abrogated in siRNA-Klotho mice (Fig. 5F), indicating that Klotho critically mediated the renal protection by HDAC inhibition. We also measured serum calcium levels and found no significant changes among groups.

### Klotho is essential for the bone protection by HDAC inhibition

**Figure 6:**
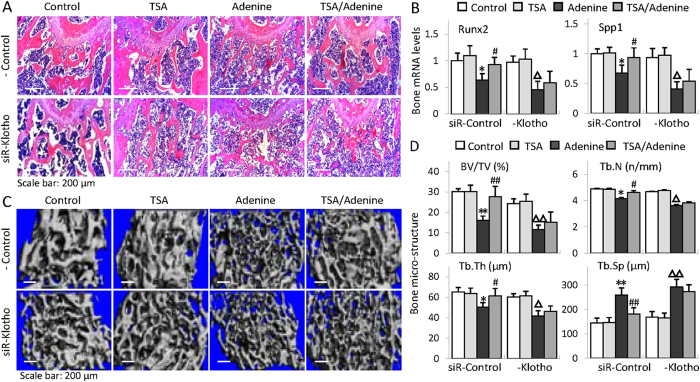
Klotho is essential for the bone protection by HDAC inhibition. (**A**) Representative H&E staining of mouse distal femur sections from siRNA-control or siRNA-Klotho-injected control, TSA, adenine and TSA-treated adenine mice (6 weeks, n = 6 in each group). (**B**) Average bone mRNA levels of Runx2 and Spp1 examined by qRT-PCR (n = 6). (**C**) Representative micro-CT 3D images of trabecular architectures of the distal femurs from mice as in Fig. 6A. (**D**) Quantitative analyses of the ratio of bone volume to tissue volume (BV/TV), trabecular number (Tb.N), trabecular thickness (Tb.Th) and trabecular separation (Tb.Sp) from micro-CT examinations (n = 6). Data are presented as the mean ± SD. *P < 0.05, **P < 0.01 versus control, ^#^*P* < 0.05, ^##^*P* < 0.01 versus adenine treatment in siR-control mice; ^Δ^*P* < 0.05, ^ΔΔ^*P* < 0.01 versus control in siR-Klotho mice.

We further examined the distal femur by H&E staining and found that TSA treatment reduced the thinner and deranged trabeculae and the enlarged bone marrow cavity in siRNA control-injected adenine mice, but lack of Klotho by siRNA interference largely eliminated the protective effects (Fig. 6A). In addition, TSA attenuation of decreased Runx2 and Spp1 was also diminished in siRNA-Klotho mice (Fig. 6B). To better quantitatively determine the changes of bone structures, we examined the femur micro-architectures by micro-CT (computed tomography) 3D analysis. The results showed that adenine mice displayed the typical osteoporotic changes such as less trabecular bone volume versus tissue volume (BV/TV), reduced trabecular bone number (Tb.N) and trabecular thickness (Tb,Th), and increased trabecular bone separation (Tb.Sp) (comparing column 1 and 3 in Fig. 6C and lane 1/4 and 3/7 in Fig. 6D) in siRNA control-injected mice, whereas TSA treatment significantly inhibited these alterations (comparing column/lane 4 and 3 upper panel in Fig. 6C and D). However the protective effects were largely abolished in siRNA-Klotho-injected mice (comparing column 4 and 3 lower panel in Fig. 6C and lane 8 and 7 in Fig. 6D). Altogether these results strongly indicate that Klotho plays a critical role in the bone protection by HDAC inhibition in CKD-MBD mice.

## Discussion

In this study we aimed to explore the potentials of Klotho restoration by epigenetic HDAC inhibition and its functional relevance to the renal and bone protections in a mouse model of CKD-MBD. We found that HDAC inhibitor TSA effectively reduced renal injury with the impressive Klotho restoration, improved the deregulated key factors involved in bone remodeling and attenuated the associated bone damage. We further demonstrated that HDAC inhibition prevented Klotho loss by increasing the promoter acetylation and its transcription. Finally we showed that the Klotho restoration by HDAC inhibition plays a critical role for the renal and bone because the protective effects were significantly reduced in mice lacking Klotho. Thus the results from our study revealed a key target and the mode of action of the kidney and bone protections by HDAC inhibition.

Adenine mouse model was initially established in rat for studying chronic renal failure and turned out to be a good model of CKD-MBD^32^,^34^,^35^. Adenine is readily converted to 2, 8-dihydroxyadenine in kidney, forming insoluble crystals that block renal tubules and subsequently causing extensive renal tubule damage, disturbed mineral and hormone metabolisms and the consequential osteodystrophy^32^,^36^. In our study, adenine-fed mice for 6 weeks develop typical renal lesions of CKD with marked increase of serum phosphorus, iPTH and FGF23, 1, 25(OH)_2_D_3_ reduction and osteoporosis, except that calcium levels stayed normal as reported previously by others^37^. More importantly, the mice showed a drastic Klotho suppression in kidney and blood circulation. Thus adenine-fed mice are ideal for studying Klotho functions and its relevance to CKD pathogenesis with bone complications.

CKD development and progression are promoted by a variety of pathological processes of various etiologies^38^. Epigenetic modulations of renal protective or pathological protein expressions by DNA and protein methylation, protein acetylation or miRNA modification add additional controls over the processes. Aberrant HDAC activities are reportedly involved in various acute and chronic kidney diseases and affect many cellular pathological processes such as inflammation and renal fibrosis. In particular, HDAC1 and 2 are associated with TNFα-incurred acute kidney injury^20^; abnormal HDAC2 and HDAC4 mediate the pathogenesis of diabetic podocytopathy^39^,^40^ and HDAC1 and HDAC6 contribute to the progression of polycystic kidney disease^41^,^42^. HDAC inhibitions with general or selective HDAC inhibitor effectively improved the pathological changes in the animal studies. However many cell components mediate CKD pathogenesis and the key genes that confer the renal protection by HDAC inhibition are incompletely understood. Now our results provided clear evidence that HDAC inhibition-associated Klotho restoration improved the disturbed mineral and hormone metabolisms, attenuated the expression of CKD-associated proteins and reduced the renal and bone injuries in adenine mice, establishing Klotho as a critical target of HDAC inhibition in the renal and bone protections.

Klotho prevents the renal and bone damage in adenine mice essentially through interrupting pro-fibrotic signaling. As demonstrated in our previous^33^ and current studies that Klotho preservation by endogenous Klotho re-activation in adenine mice inhibited TGFβ- induced Smad signaling (Figs 2B and 5D), therefore reducing myofibroblast trans-differentiation and extra cellular matrix protein expressions. Klotho beneficial modulations of disturbed mineral and FGF23 metabolisms^33^ (Fig. 2 in this study) as well as its anti-inflammation^43^ and oxidative stress-balancing^44^ functions presumably provide additional protections. BMP-7 is another renal and bone protective protein and its down-regulation by aberrant HDAC activities worsen renal and bone pathogenesis^45^. BMP-7 plays pivotal roles promoting the proliferation and repair of the tubular cells after renal injury and beneficially affects bone remodeling by promoting osteoblast growth and differentiation^46^,^47^. Thus, BMP-7 recovery by HDAC inhibition in adenine mouse kidney as demonstrated in our study (Fig. 2B) also contributes to the renal and bone protections. Intriguingly, BMP-7 recovery by HDAC inhibition in adenine mouse kidney is causally affected by Klotho (Fig. 5), highlighting the essential role of Klotho restoration in the renal and bone protections by HDAC inhibition.

Bone undergoes continues remodeling balanced by two opposite processes - the osteoblast maturation-associated bone formation and the osteoclast-mediated bone resorption. It is reported that HDAC inhibition by TSA promotes osteoblast maturation *in vitro*, inhibits osteoclast differentiation of bone marrow cells^48^,^49^ and mitigated osteoporotic injuries in animal studies^50^. Interestingly, previous studies reported that bone expressed low levels of Klotho^51^,^52^ and we found that TSA also substantially recovered the Klotho loss in adenine mouse femurs, which likely exerting a local protective effects. Still, kidney is the principal organ mediating Klotho effects^53^ because mice with systemic or nephron-specific Klotho knockout exhibit similar low bone formation activities and osteopenia^8^,^17^,^53^, suggesting that renal Klotho directly regulates the different stages of bone formation, remodeling and repair. In addition, some key minerals and hormones affected by Klotho such as calcium, phosphorus, FGF23 and PTH are essential for bone homeostasis^5^. Our results showed that HDAC inhibition by TSA not only effectively preserved Klotho, improved the CDK-associated protein expressions and reduced the renal pathogenesis in CKD-MBD mice, but also Klotho-sensitively normalized the key factors that causally affecting kidney and bone remodeling and consequently alleviated the kidney and bone damage, supporting that Klotho preservation contributes, at least in a significant part, to the kidney and bone protections by HDAC inhibition, which is consistent with previous study indicating that endogenous Klotho restoration by chemicals attenuated CKD-associated extrarenal complication of cardiovascular calcification^18^.

HDAC aberration-associated Klotho repression has been observed in TNFα-mediated NF-kB signaling in renal cells, in which HDAC1 and HDAC2 physically interact with NF-kB upon TNFα stimulation and presumably assist the transcriptional down-regulation of Klotho^20^; however this mode of action has not been verified *in vivo*. The promoter hypermethylation-mediated Klotho repressions are found in patients and animal models of CKD^30^,^54^. DNA methylation modification adds a methyl group to the cytosine residue within cytosine- phosphate-guanine (CpG) islands often located on gene’s enhancer or promoter^55^, which serves as a docking site for transcriptional repressor and HDAC to transcriptionally repress gene expression^56^,^57^, therefore requiring HDAC activities. Klotho promoter contains a large stretch of GC islands and binding sites for NF-kB^20^ and PPARγ^58^. We demonstrated that HDAC inhibition-associated Klotho preservation occurred at the transcription levels and Klotho promoter is associated with reduced acetylation of histone3 in adenine mouse kidney, but HDAC treatment effectively inhibited the reduction, suggesting that TAS preserved Klotho through a transcriptional up-regulation process. Epigenetic modifications of histone and DNA involve complicated and site-specific interplay with accessory mediators. Our results provide a molecular basis for the observed Klotho up-regulation by HADC inhibition and help explore the precise underlying mechanisms and the therapeutic designs.

In conclusion, the results from our study represent the first *in vivo* functional evaluation of the therapeutic efficacy of HDAC inhibition on Klotho restoration and CKD-MBD pathogenesis in mice. In addition, our results opened up new perspectives for the development of Klotho-targeted therapy in treating CKD and the associated extrarenal complications.

## Materials and Methods

### Animals and experimental design

Adenine model of CKD-MBD was established with C57BL/6 male mice of 8-weeks of age according to a previously-established protocol^33^. Mice were randomly divided into Control, TSA (SelleckChem, USA), Adenine (Sigma-Aldrich, USA) and TSA-treated Adenine groups (n = 6) and the experiments went for 6 weeks. Control mice were fed a standard powder diet containing 1.16% calcium, 0.73% phosphate, 18.2% protein, and 7.56 IU/g vitamin D3, (Collaboration BioMedical Inc., Nanjing, China); TSA mice received intraperitoneal injection of TSA (0.5 mg/kg body weight in 100 μl of PBS) daily; Adenine mice were fed the regular diet containing 0.2% adenine. At sacrifice, kidney, blood and femur were collected and stored at –80 °C until analysis. Use of animal and the experimental protocol were in accordance with the University Guidelines and approved by the Institutional Animal Care Committee (IACUC) of Nanjing University Medical School.

### siRNA-mediated Klotho knockdown in mouse study

Mice were divided into two groups receiving siRNA-Klotho (targeting 5′-GCGACTACCCAGAGAGTAT-3′ in mouse Klotho gene)^59^ or a scrambled RNA 5′-CGUACGCGGAAUACUUCGA-3′ as control, respectively. Each mouse received 6 siRNA injections (10 nm in 200 μl of PBS) through tail vein once a week before and during adenine feeding for a total of 6 weeks. Both groups were subjected to TSA, adenine or TSA plus adenine treatment as above (n = 6 in each group).

### Serum markers, histomorphological and histoimmunochemistry assay

Serum BUN, creatinine, calcium, and phosphorus were measured using a multichannel autoanalyzer (Hitachi 7180, Hitachi Ldt, Japan). Serum intact PTH (iPTH), FGF23 (C-terminal, Immutopics, USA), 1,25-dihydroxyvitamin D3 (1,25 (OH)_2_D_3_ (Immunodiagnostic Systems, UK) and Klotho (SEH757Mu, USCN Life Science Inc., China) were measured using enzyme-linked immunosorbent (ELISA) assay kits following manufacturers’ protocols.

Kidney tissue sections (3 μm) and femur sections (10 μm) were prepared by standard protocols and stained with either Mason’s trichrome staining or H&E (hematoxylin and eosin) method^33^,^60^. Immunohistochemistry was performed using anti-Klotho antibody. The images were obtained using a Nikon E800 microscope. The renal fibrosis was calculated as the ratio of collagen deposition in renal cortex (blue color area in Masson’s trichrome-stained sections) over the whole cortex area and semi-quantitatively measured from 10 randomly selected fields of each section of all experimental animals and analyzed by Image J software.

### Bone mineral density and trabecular microstructure analyses

Bone mineral density (BMD) of femurs was measured with a Faxitron MX20 Specimen Radiography System (Faxitron X-ray Corp.,USA) and analyzed by Image J software. For trabecular micro-structure analysis, femurs were scanned with a micro-computed tomography scanner VivaCT80 (SCANCO Medical, Switzerland) at 15.6 μm voxel size, with 250 ms integration time, 55 kVp energy, and 145 μA intensity. A total of 100 slices from each sample were used to analyze the micro-architectures of trabeculae at the distal metaphyses, 78 μm from the growth plate. Three Dimensional (3D) images of trabeculae were acquired and the ratio of bone volume to tissue volume (BV/TV), trabecular number (Tb.N), trabecular thickness (Tb.Th) and trabecular separation (Tb.Sp) were calculated.

### Cell culture

Human proximal tubular epithelial cells (HK-2) were obtained from American Type Cell Culture (ATCC. USA), cultured in DMEM F-12 (Gibco, USA) medium supplemented with 10% fetal bovine serum (Gibco, USA) and maintained in a 5% CO_2_ incubator at 37 °C.

### Klotho promoter reporter assay

The mouse Klotho promoter reporter plasmid containing 2 kb upstream of the transcription starting site (mKLp-Luc) constructed by PCR amplification of genomic DNA from mouse RAW cells in pGL3-basic vector has been described^33^. For luciferase assay, the reporter and a renilla luciferase control plasmids were co-transfected into HK2 cells with Lipofectamine 2000 reagents (Invitrogen, USA). Twenty four hours later, TSA was added for additional 24 hour and the cell lysates were collected and the luciferase activities were measured with a Luciferase Reporter Assay System (Promega, USA) in a GloMax luminometer. The relative luciferase activities were calculated as the ratio of luciferase activities of the reporter divided by the renilla activities.

### Western blotting

Western blotting was performed with HK2 cells treated with various doses of TSA for different times, or with kidney tissue lysates essentially as before^61^. Aliquots of protein were subjected to sodium dodecylsulfate–polyacrylamide minigel electrophoresis and transferred to a Hybond-P membrane (GE Healthcare, Little Chalfont, UK), and then incubated with following primary and secondary antibodies: anti-Klotho (KO603, TransGenic, Japan), α-SMA (Abcam, MA, USA), E-cadherin (BD Transduction Laboratories, San Jose, USA), BMP-7 (Bioworld, USA), Phosphorylated Smad3 (Cell signaling Biotech, USA), acetyl-histone H3 (Millipore, Billerica, USA), β-actin, goat anti-rabbit IgG-HRP, and goat anti-mouse IgG-HRP (Yifeixue Biotech, Nanjing, China). Chromogenic detection was performed with ECL Western Blotting Detection Kit and quantified using Image J Software.

### Quantitative real-time polymerase chain reaction (qRT-PCR)

Total RNA from femur and kidney was extracted using TRI reagent (Sigma-Aldrich, USA).cDNA was synthesized from 2 mg of total RNA using a transcript first-strand cDNA synthesis Kit (Vazyme, China). The qRT-PCR reactions were performed in triplicate using the SYBR Green PCR Master Mix (Vazyme, China) with primer mKlothoF (5′-GATGGCAGAGAAATCAACACAGT-3′) and mKlothoR (5′-ACTACGTTCAAGTGGACACTACT-3′), Runx2 F: 5′-GACTGTGGTTACCGTCATG GC-3′; Runx2 R: 5′-ACTTGGTTTTTCATAACAGCGGA-3′; Spp1F: 5′-ATCTCAC CATTCGGATGAGTCT-3′; Spp1R: 5′-TGTAGGGACGATTGGAGTGAAA-3′.

### Chromatin immunoprecipitation (ChIP)

Chromatin immunoprecipitation assay (ChIP) was carried out with a ChIP assay kit (Millipore, Billerica, MA) according to the manufacturer’s instructions. The Immunoprecipitation was performed with anti-acetyl histone H3 antibody (Millipore, Billerica, MA) or an isoform-matched IgG as control. Immunoprecipitated DNA was further PCR-amplified using primer set specific for mouse Klotho promoter mKLpF (5′-GCTGAGTTGTACCTTACTGAG-3′) and mKLpR (5′-CACCATATCCCGTTCATCAC-3′). PCR amplification profiles: 94 °C for 5 min followed by 94 °C 30 sec, 55 °C 1 min, 72 °C 1 min for a total of 30 cycles and a final 72 °C for 10 min. The PCR products were analyzed on 1.5% agarose gel and visualized under UC light.

### Statistical analysis

Data are represented as mean ± standard deviation (SD). Statistical differences were assessed by Student’s *t*-test for comparisons of two groups, one-way analysis of variance (ANOVA) or ANOVA followed by Tukey’s post hoc test for comparisons of multiple groups. *P* < 0.05 and *P* < 0.01 were considered statistically significant and very significant.

## Additional Information

**How to cite this article**: Lin, W. *et al*. Klotho restoration via histone deacetylase inhibition attenuates chronic kidney disease-associated bone injury in mice. *Sci. Rep.* **7**, 46195; doi: 10.1038/srep46195 (2017).

**Publisher's note:** Springer Nature remains neutral with regard to jurisdictional claims in published maps and institutional affiliations.
